# Supplementary material for: Stability and nature of the volume collapse of ε-Fe2O3 under extreme conditions
Source: Nat Commun. 2018 Nov 1;9:4554. doi: 10.1038/s41467-018-06966-9 (PMC6212538; doi:10.1038/s41467-018-06966-9)
Supplement: Supplementary file 1 — Supplementary Information [file 41467_2018_6966_MOESM1_ESM.pdf]

Supplementary Information of "Stability and nature of the volume collapse of ε-Fe_2O_3 under extreme conditions"

J. A. Sans,[1,] V. Monteseguro,[2,3] G. Garbarino,[2] M. Gich,[4] V. Cerantola,[2] V. Cuartero,[2,5] M. Monte,[2] T. Irifune,[6,7] A. Muñoz[8] and C. Popescu[9]*

[1] Instituto de Diseño para la Fabricación y Producción Automatizada, MALTA Consolider Team, Universitat Politècnica de València, 46022 Valencia, Spain
[2] European Radiation Synchrotron Facility 38043 Grenoble Cedex 9, France
[3] ICMUV. MALTA Consolider Team, Universitat de València, 46100 Burjassot, Spain
[4] Institut de Ciència de Materials de Barcelona (ICMAB-CSIC), 08193 Bellaterra, Spain
[5] Centro Universitario de la Defensa de Zaragoza. Ctra. Huesca s/n, 50090 Zaragoza, Spain.
[6] Ehime University, 2–5 Bunkyo-cho, Matsuyama 790-8577, Japan
[7] Earth-Life Science Institute, Tokyo Institute of Technology, Tokyo 152-8500, Japan
[8] Departamento de Física, Instituto de Materiales y Nanotecnología, MALTA Consolider Team, Universidad de La Laguna, 38207 San Cristóbal de La Laguna, Spain
[9] ALBA-CELLS, 08290 Cerdanyola del Vallés, Barcelona, Spain

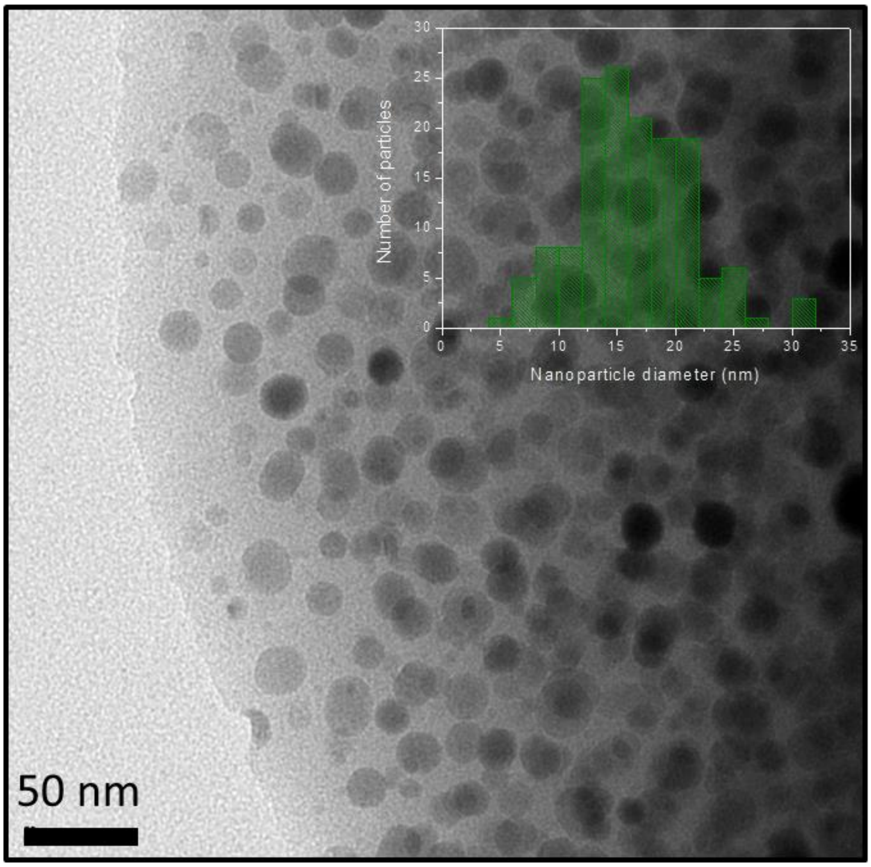

Supplementary Figure 1. Electron microscopy of ε-Fe_2O_3 nanoparticles studied here. (inset) Statistical distribution of the nanoparticles size.

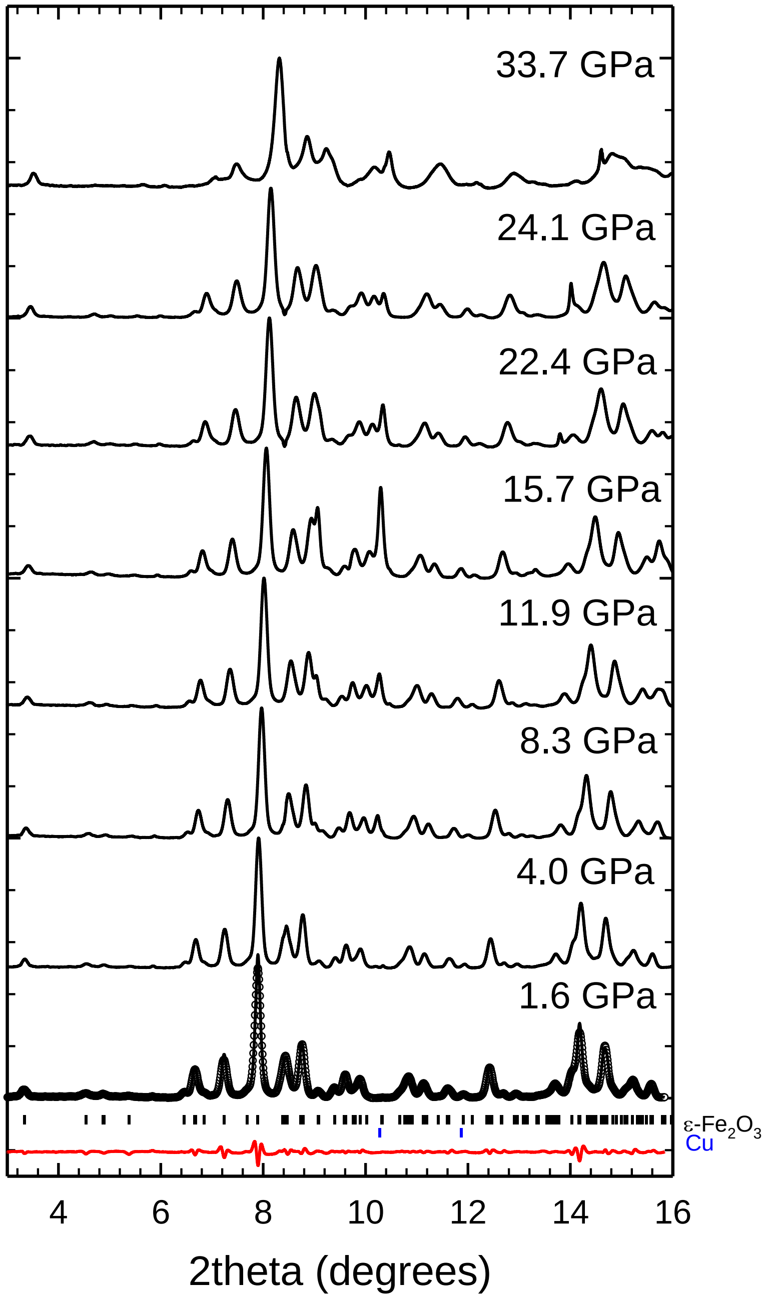

Supplementary Figure 2. X-ray diffraction patterns of ε-Fe_2O_3 nanoparticles (red) at several pressures, vertically shifted for the sake of clarity.

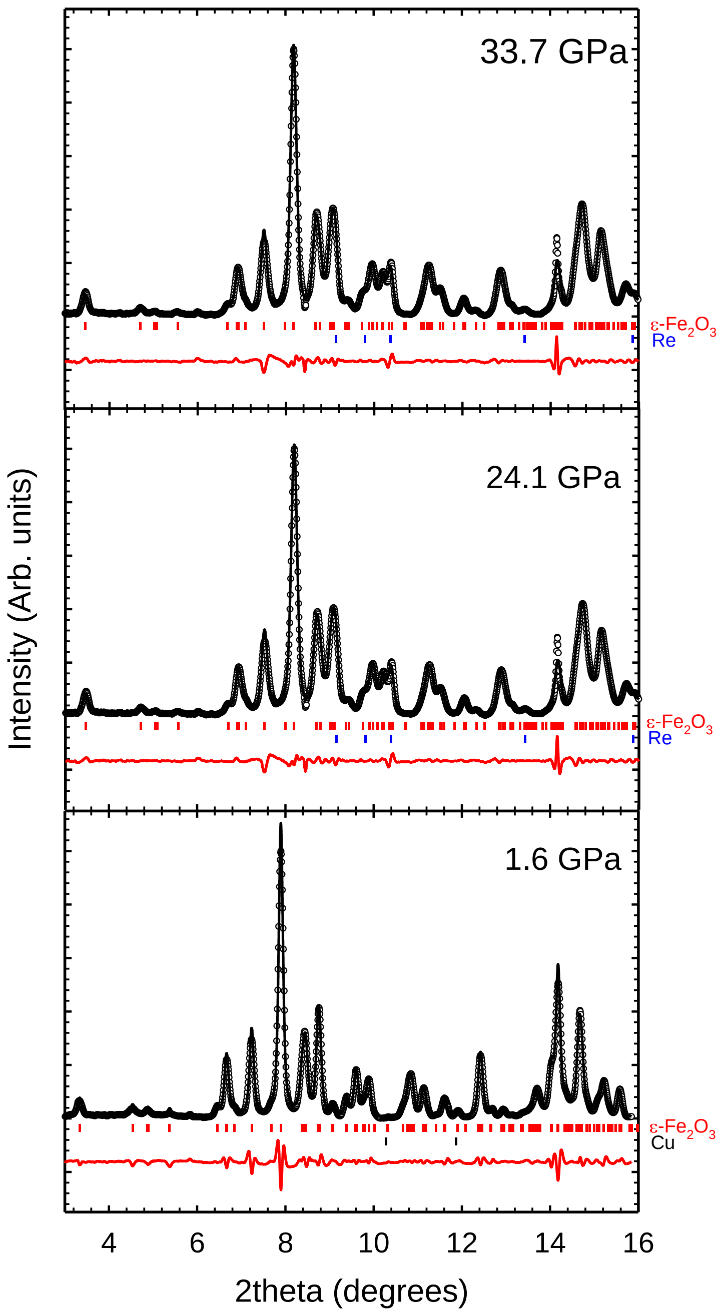

Supplementary Figure 3. Rietveld Refinement (black line) of the experimental x-ray diffraction patterns (symbol) collected at several pressures. Above 2 GPa, it was observed rhenium coming from the gasket (blue vertical ticks). Residuals (red line) are plotted to show the quality of the fitting.

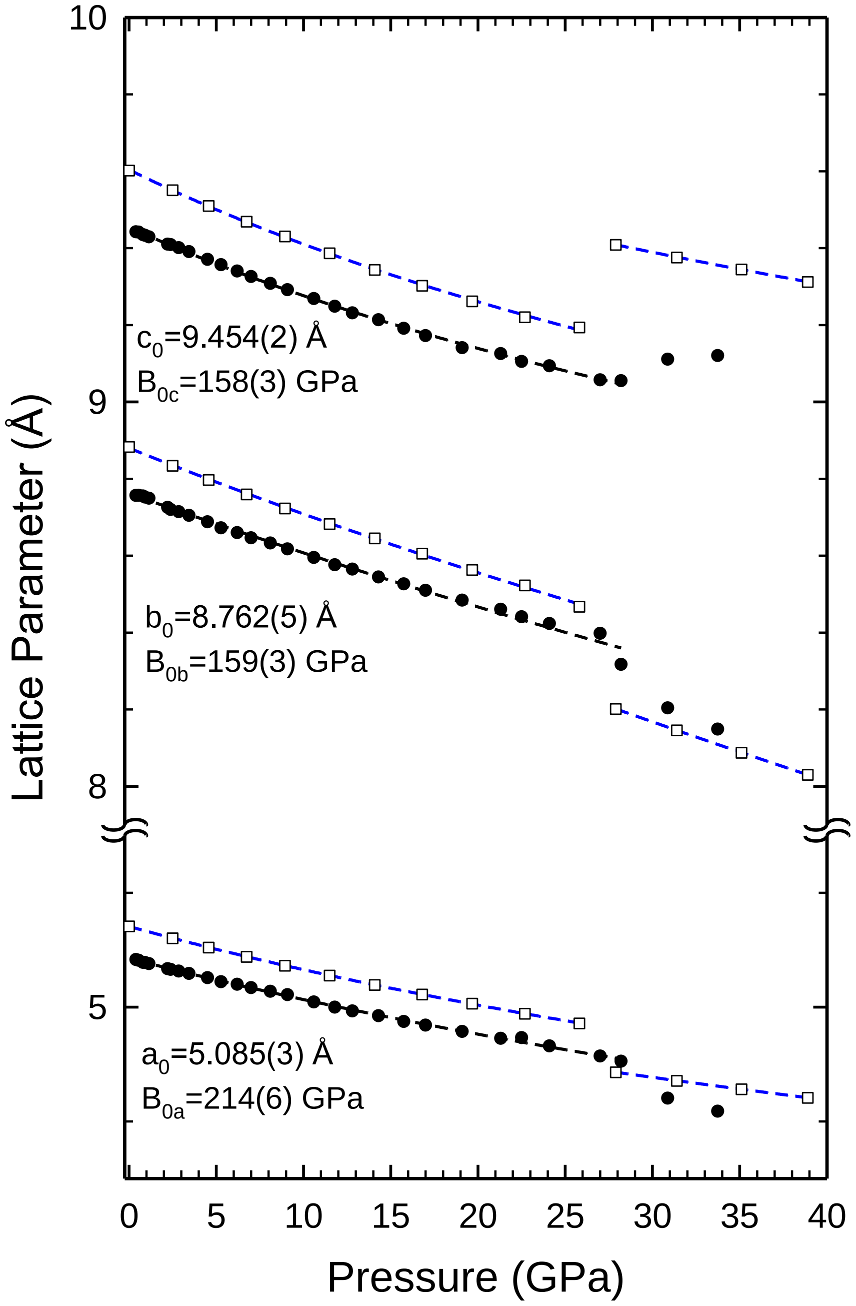

Supplementary Figure 4. Evolution of the experimental (black symbols) and theoretically simulated (empty squares) lattice parameters under pressure.

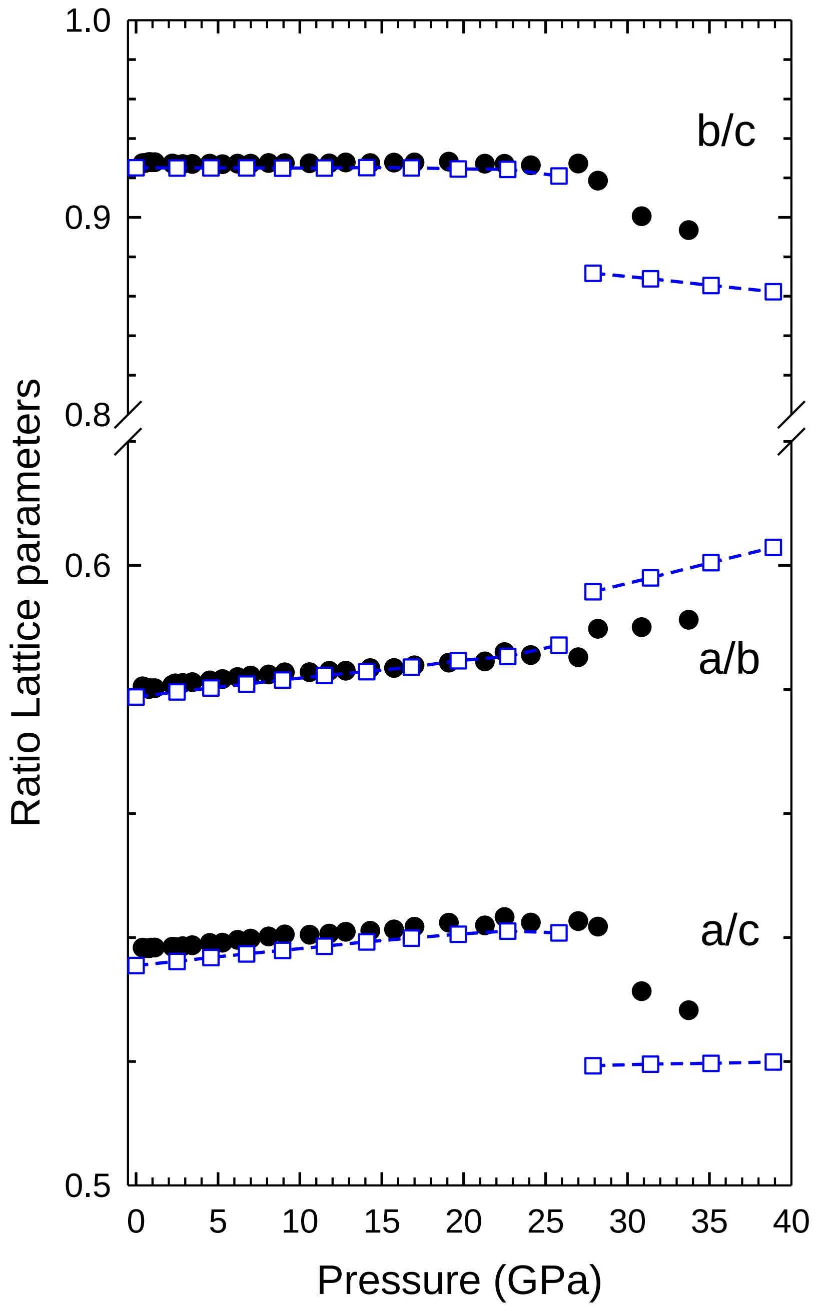

Supplementary Figure 5. Evolution of the experimental (black symbols) and theoretically simulated (empty squares) ratio between the lattice parameters of compressed ε-Fe_2O_3.

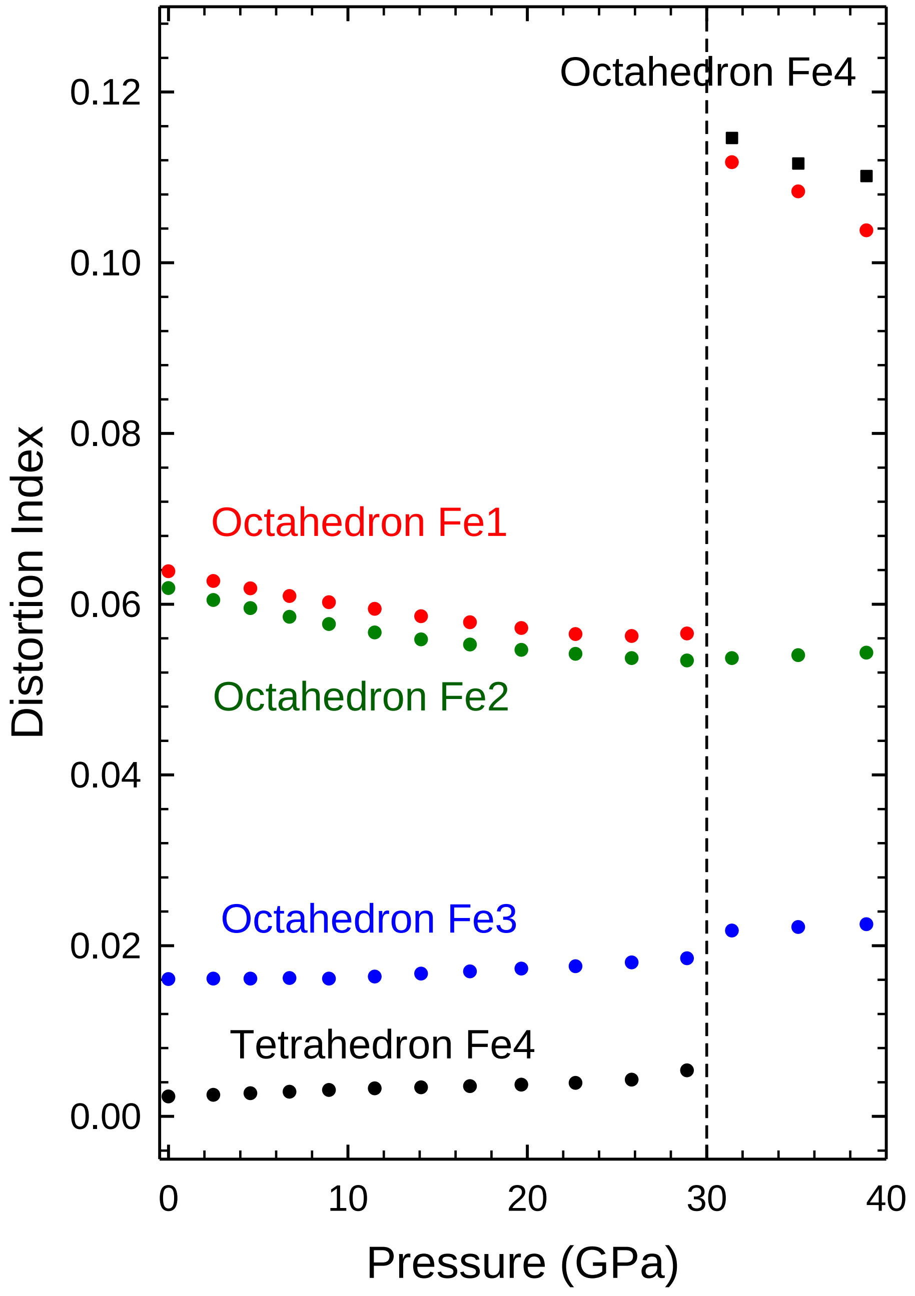

Supplementary Figure 6. Evolution of the theoretically simulated values of the distortion index obtained from compressed ε-Fe_2O_3.

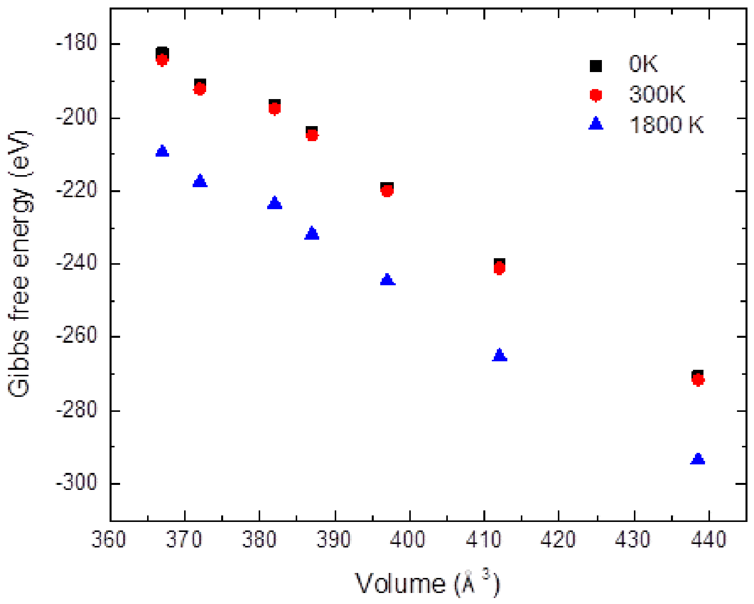

Supplementary Figure 7. Gibbs free energy vs volume for ε-Fe_2O_3 at 0K, 300K and 1800K

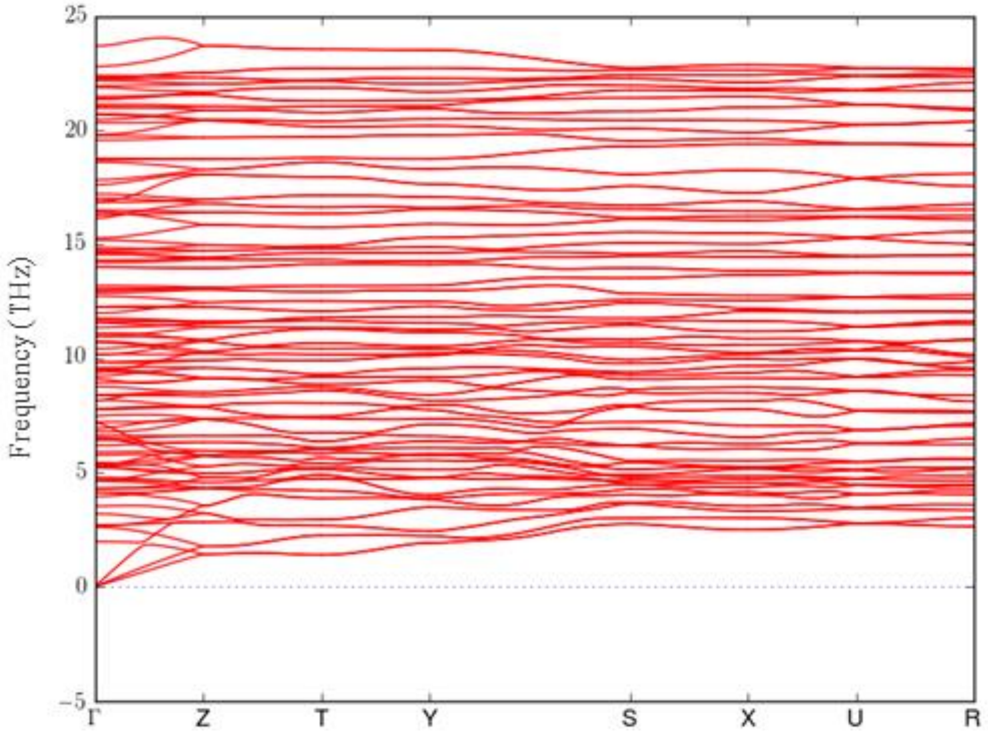

Supplementary Figure 8. Phonon dispersion curve of ε-Fe_2O_3 at 25 GPa and 1800K.
